# Automatic detection of valve events by epicardial accelerometer allows estimation of the left ventricular pressure trace and pressure–displacement loop area

**DOI:** 10.1038/s41598-020-76637-7

**Published:** 2020-11-18

**Authors:** Ali Wajdan, Magnus Reinsfelt Krogh, Manuel Villegas-Martinez, Per Steinar Halvorsen, Ole Jakob Elle, Espen Wattenberg Remme

**Affiliations:** 1 The Intervention Centre, Oslo University Hospital, Rikshospitalet, Oslo, Norway; 2 Department of Informatics, University of Oslo, Oslo, Norway; 3 Institute of Clinical Medicine, Faculty of Medicine, University of Oslo, Oslo, Norway; 4 Institute for Surgical Research, Oslo University Hospital, Rikshospitalet, Oslo, Norway

**Keywords:** Cardiology, Health care

## Abstract

Measurements of the left ventricular (LV) pressure trace are rarely performed despite high clinical interest. We estimated the LV pressure trace for an individual heart by scaling the isovolumic, ejection and filling phases of a normalized, averaged LV pressure trace to the time-points of opening and closing of the aortic and mitral valves detected in the individual heart. We developed a signal processing algorithm that automatically detected the time-points of these valve events from the motion signal of a miniaturized accelerometer attached to the heart surface. Furthermore, the pressure trace was used in combination with measured displacement from the accelerometer to calculate the pressure–displacement loop area. The method was tested on data from 34 animals during different interventions. The accuracy of the accelerometer-detected valve events was very good with a median difference of 2 ms compared to valve events defined from hemodynamic reference recordings acquired simultaneously with the accelerometer. The average correlation coefficient between the estimated and measured LV pressure traces was r = 0.98. Finally, the LV pressure–displacement loop areas calculated using the estimated and measured pressure traces showed very good correlation (r = 0.98). Hence, the pressure–displacement loop area can be assessed solely from accelerometer recordings with very good accuracy.

## Introduction

Monitoring of cardiac function by miniaturized accelerometers attached to the heart is becoming more frequently used, for example, these sensors can be found in pacing electrodes for cardiac resynchronization therapy^1–3^. Similarly, accelerometers may also be incorporated in the temporary pacemaker leads that are attached to the heart during cardiac surgery and later retracted through the chest after a few days. Addition of such a sensor to the pacemaker lead will allow measurements of cardiac motion without adding complexity to the surgical procedure, thus providing a new method for continuous monitoring of cardiac function in these patients. This can be useful for assessing the response to medication and may lead to earlier detection of cases with myocardial dysfunction.

**Figure 1:**
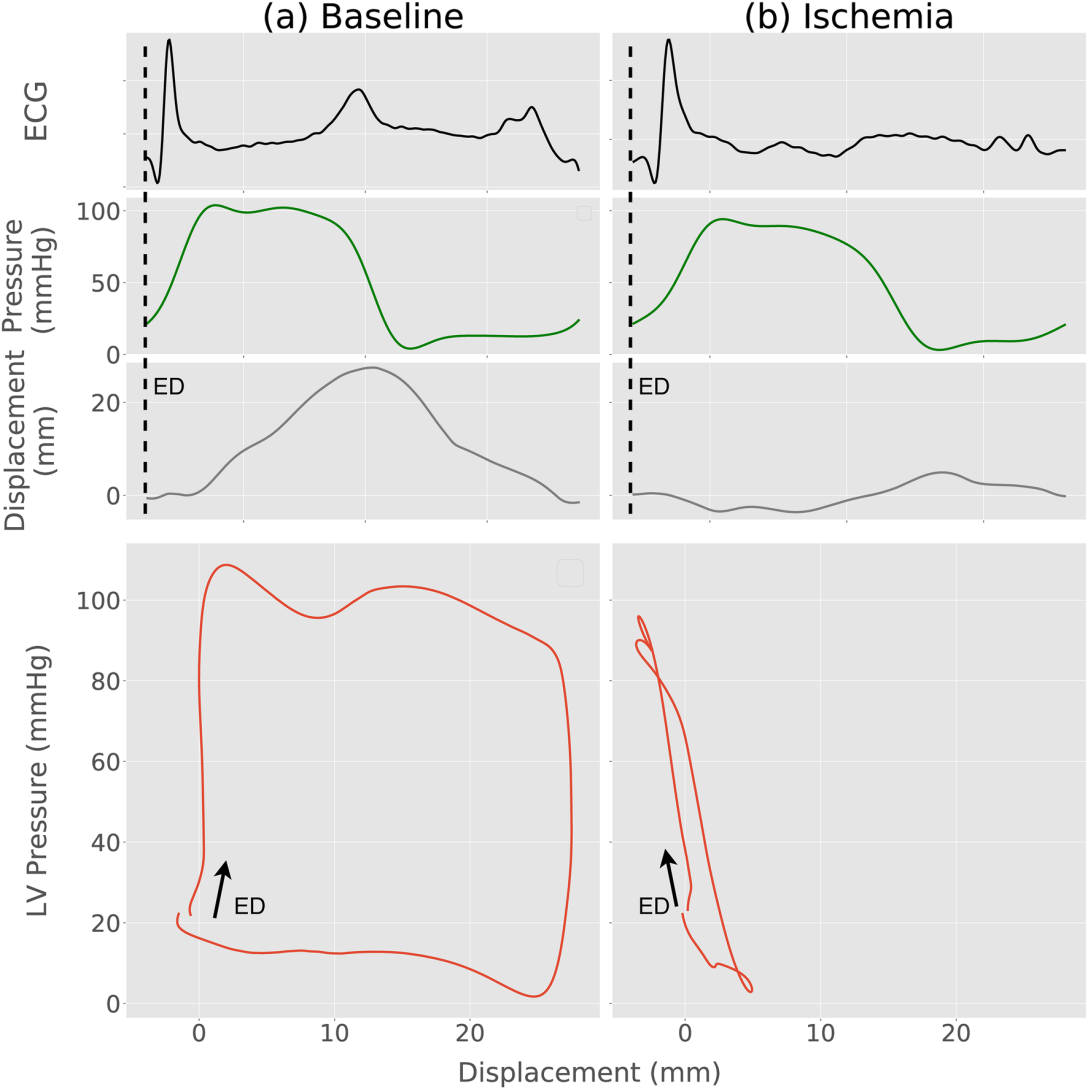
Pressure–displacement loops at **(a)** baseline and (**b)** after induction of ischemia, in one animal. The arrow indicates the loop direction from the end diastolic (ED) time-point.

In order to assess cardiac function by using accelerometers, functional indices need to be extracted from the sensor signal. Our group has performed several studies with epicardially attached accelerometers and demonstrated that such accelerometer recordings can be used for monitoring cardiac function^4,5^. Velocity and displacement, calculated by integrating the acceleration signal once and twice, respectively, have been shown to give valuable functional information^4,6^. Another functional index can be derived by combining displacement with measurements of left ventricular (LV) pressure to generate pressure–displacement loops. This builds on the classical pressure–volume loop principle for analysis of cardiac function which also has inspired other novel loop-analysis methods such as the pressure-strain loop method^7,8^. In a previous study we showed that the pressure–displacement loop area had very high sensitivity and specificity for detection of ischemia in a pig model^9^. In a normal heart, displacement is well coordinated with pressure so a point on the heart wall moves in one direction during ejection and back during filling, creating a large, open pressure–displacement loop (Fig. 1a). Conversely, during myocardial ischemia, there is typically a paradoxical motion pattern which results in less coordinated displacement. Hence, a point on the heart wall may move in the opposite direction at the beginning of systole before it subsequently starts moving in the normal systolic direction resulting in a distorted pressure–displacement loop with an effectively smaller loop area (Fig. 1b).

Pressure–displacement loops may be a good functional parameter, but it requires invasive measurements of the ventricular pressure which is not performed in clinical practice due to stroke risk. Furthermore, the invasiveness of the procedure carries risks derived from the arterial puncture and left heart catheterization such as bleeding, tamponade, and/or arrhythmias. However, Russell et al*.*^10^ proposed a noninvasive method to estimate the LV pressure trace and generate a pressure-strain loop with high accuracy. Briefly described, a normalized, averaged LV pressure trace can be made patient specific by scaling its peak pressure to the patient’s systolic brachial cuff pressure, and by time-adjusting the isovolumic, ejection, and filling phases to the measured time-points of closure and opening of the mitral and aortic valves. The time-points of the valve events were manually assessed by echocardiography in that study.

In this study we hypothesize that an accelerometer attached to the LV epicardium can be used to automatically detect these valve events as each of these events corresponds to a distinct oscillation pattern in the acceleration signal. Previous studies^11–13^ with an accelerometer attached on the skin of the chest have shown that vibrations on the skin correlate with the opening and closing of heart valves. Furthermore, we hypothesize that such automatic valve event detection can be used to estimate the individual LV pressure trace as proposed by Russell et al*.*^10^, which subsequently can be used to generate the pressure–displacement loop based solely on accelerometer recordings without the need for invasively measured ventricular pressure.

To test the hypotheses, we first developed a signal processing method for automatic detection of the valve events from the acceleration signal. The timing of each accelerometer-detected valve event was compared with the hemodynamic defined reference points in a cohort of animals where such extensive hemodynamic measurements had been recorded. Lastly, in a retrospective analysis of a larger cohort of animals with less extensive hemodynamic measurements, the accuracy of the accelerometer-based estimation of the pressure trace and pressure–displacement loop area were validated.

## Methods

### Animal preparation

**Figure 2:**
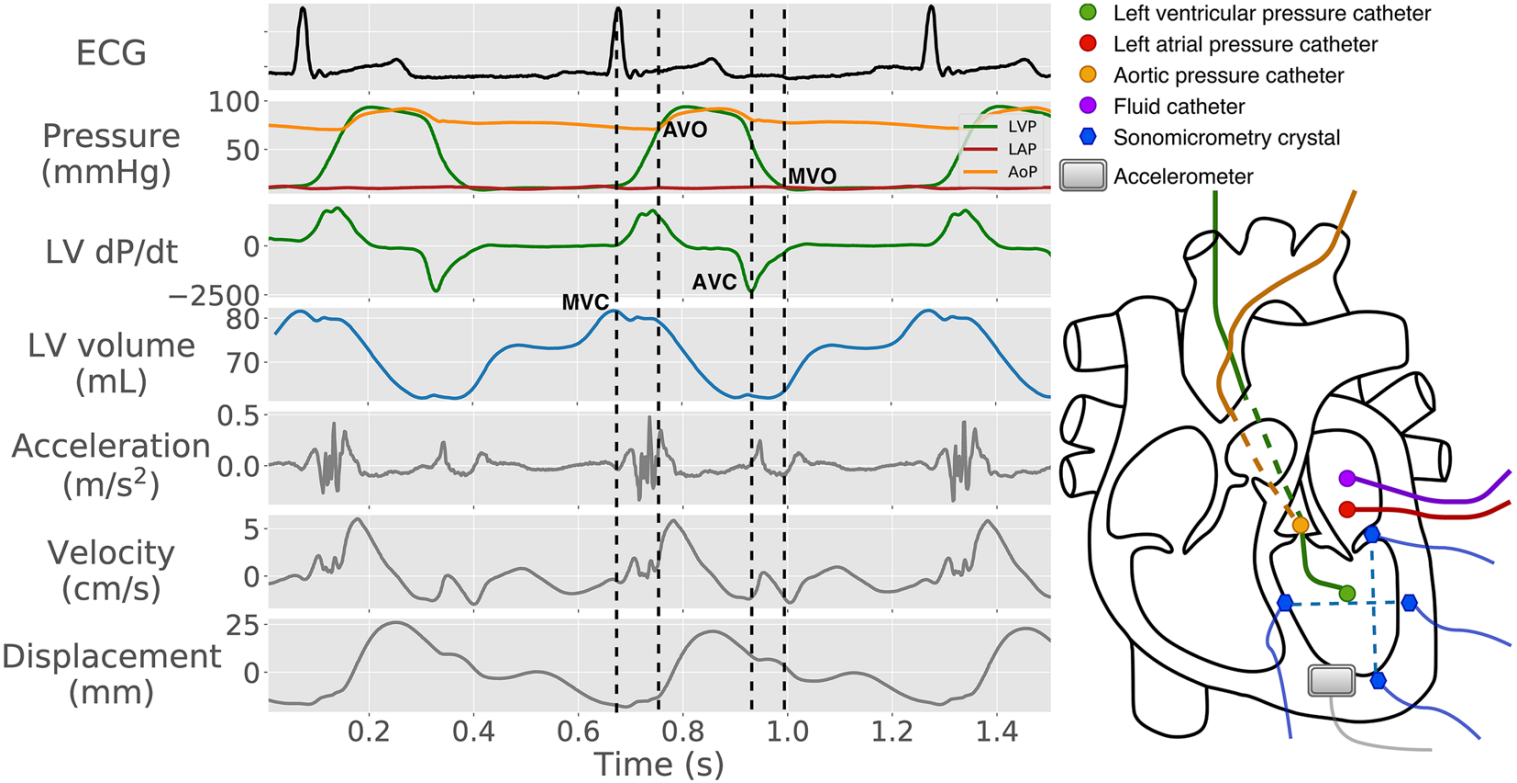
ECG and hemodynamic recordings from a representative canine experiment are shown in the left panel with circumferential acceleration and calculated velocity and displacement. Dashed, vertical lines represent the reference points marking the opening and closing of the mitral and aortic valves. The right panel shows a schematic of the instrumentation. *MVC *mitral valve closure, *AVO* aortic valve opening, *AVC* aortic valve closure, *MVO* mitral valve opening.

Ten mongrel canines (36 ± 4 kg SD) of either sex were used for validation of the accelerometer-based detection of valve events. The accelerometer measurements were added to the protocol of a previous study as well as an ongoing study at Oslo University Hospital. The protocols were approved by the Norwegian Food Safety Authority [project ID: 8628 and 17644] and carried out in accordance with Norwegian regulations concerning use of animals in experiments. The animals were ventilated and surgically prepared as previously described^14,15^. In short, these were acute, open chest experiments. Calibrated micromanometer-tipped catheters (MPC-500, Millar Instruments Inc, Houston, TX) were inserted into the LV, aorta, and left atrium (LA) (Fig. 2). The aortic pressure catheter was positioned immediately proximal to the aortic valve to avoid the delay from aortic valve opening to aortic pressure rise which occurs distally in the aorta. The LV and LA micromanometers were drift-adjusted relative to a fluid-filled catheter in the LA, using the average pressure of the long diastasis of post-extra-systolic beats induced at the end of each recording. Three pairs of sonomicrometric crystals (Sonometrics Corporation, London, Ontario, Canada) were implanted in the LV subendocardium to measure the LV long-axis diameter, septal-to-lateral wall diameter, and anterior–posterior diameter. LV volume was calculated using an elliptical formulation equal to the product of π/6 and the three diameters^16^. A three-axis accelerometer sensor (MPU9250, InvenSense Inc, San Jose, CA, USA) was sutured to the epicardium in the LV apical, anterior region. The x-, y-, and z-axis of the accelerometer were aligned with the longitudinal, circumferential, and radial directions, respectively. ECG, pressures, sonomicrometry, and accelerometer data were recorded simultaneously, accelerometer data at 650 Hz and the other data at 200 Hz.

Additional data from previous studies in 24 porcines (44 ± 4 kg SD) were used for validation of the accuracy of the accelerometer-based method for estimations of the LV pressure trace and pressure–displacement loop. These studies included measurements from 15 open chest^9,17^ and 9 closed chest porcines^18^, the latter included to test the method also under closed chest conditions. The animals were anesthetized and surgically prepared as previously described^17^. These were also acute experiments where ECG, LV pressure, and accelerometer measurements were recorded simultaneously in a similar manner as described above at 250 Hz. The studies were approved by the Norwegian Animal Research Authority and the Norwegian Food Safety Authority [project ID: 9303]. The protocol of these porcine studies did not incorporate measurements of LA pressure or verified proximal positioning of the aortic pressure catheter. The data from these experiments were therefore not used in the sub-study to validate the accuracy of the valve event detection algorithm.

### Experimental protocol

In the canine experiments, data were obtained from 3 different settings: baseline, induction of anterior-septal ischemia, and induction of left bundle branch block (LBBB). Anterior-septal ischemia was induced by temporary proximal left anterior descending coronary artery (LAD) occlusion. LBBB was induced by radiofrequency ablation of the left bundle branch^19,20^. Not all interventions were performed in all animals due to differences in protocols. A total of 20 recordings: from baseline (n = 10), ischemia (n = 5) and LBBB (n = 5) were analyzed. Data were recorded with the ventilator temporarily switched off to avoid respiratory artifacts.

In the open chest porcine experiments, data were obtained from five different settings: baseline, infusion of adrenaline (epinephrine, 10 µg), infusion of beta blocker (esmolol, 100 mg), infusion of vasodilator (niprid, 0.1 mg), and ischemia induced by LAD occlusion. In closed chest porcine experiments, data were obtained during closed chest baseline, fluid loading (~ 10% increase in end diastolic volume), and phlebotomy (~ 10% reduction in end diastolic volume). Data were recorded during open chest baseline in 8 out of the 9 closed chest porcines as well^21^.

### Data analysis

All signal processing and analyses were performed using Python 3.6^22^. Data covering at least 20 heart beats were used for the measurements in each recording for canines and at least 10 beats for porcines. ECG R-peaks were automatically detected using the Python package BioSPPy^23^.

#### Determination of valve events from hemodynamic measurements

The reference time-points for the valve events were determined from the hemodynamic measurements as follows (Fig. 2):

Mitral valve closure (MVC): Time of maximum LV volume.

Aortic valve opening (AVO): Time point of first increase in aortic pressure during systole.

Aortic valve closure (AVC): Time point of minimum LV dP/dt.

Mitral valve opening (MVO): Time of first diastolic crossover of LA and LV pressures.

#### Automatic detection of valve events from accelerometer measurements

**Figure 3:**
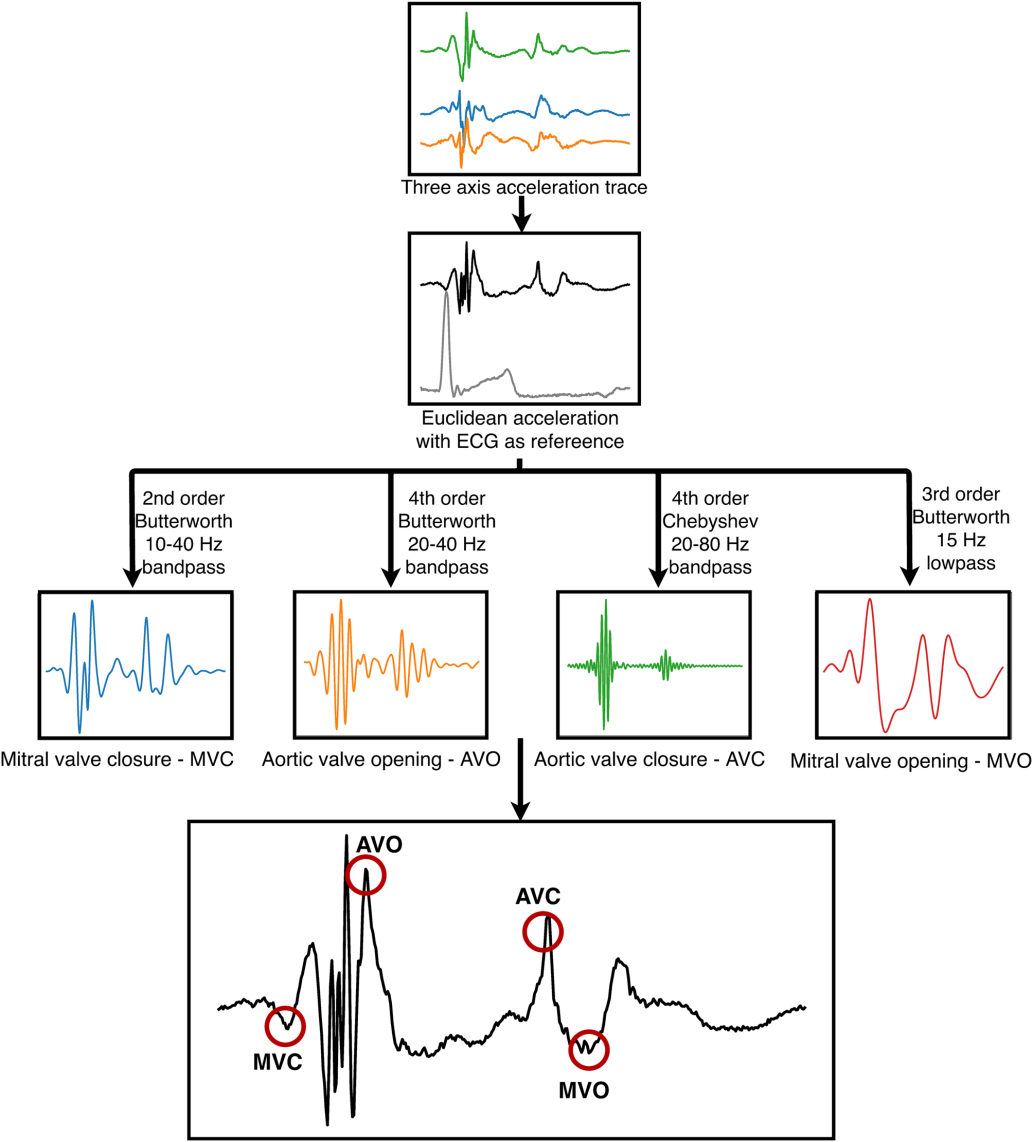
A flow chart of the algorithm used to detect the different valve events from the acceleration signal.

The valve events were automatically detected from the accelerometer signal as follows (Fig. 3): A 3-point moving average filter to smooth out any unwanted artifacts and spikes was applied to the signal of all three axes. For porcine experiments, a high-pass filter of 1 Hz was applied to remove respiratory motion artifacts, as the respiration rate was set to 15–20 breaths per minute and heart rate exceeded 60 bpm in these experiments. The accelerations recorded in the three directions (a_x_, a_y_, a_z_), was dependent on the orientation of the sensor and showed different motion patterns. The Euclidean acceleration trace (a_euclid_ = $\sqrt{{a}_{x}^{2}+{a}_{y}^{2}+{a}_{z}^{2}}$) was therefore calculated to remove this dependency and simplify the analysis. Automatically detected ECG R-peaks were used to segment the Euclidean accelerometer trace into individual heart beats. Then each event was detected as follows:

Mitral valve closure: A search window with duration of 15% of the heart cycle and starting prior to the ECG R-peak was used to locate the point of interest. A 2nd order Butterworth filter with a band pass of 10–40 Hz was applied and the first dip located using a peak/valley algorithm was determined as MVC.

Aortic valve opening: A search window with duration of 15% of the heart cycle and starting at the ECG R-peak was used. A 4th order Butterworth filter with a band pass of 20–40 Hz was applied and the highest peak detected using a peak detection algorithm was determined as AVO.

Aortic valve closure: A search window with duration of 35% of the heart cycle and starting at the detected aortic valve opening point was used. A 4th order Chebyshev bandpass filter with a pass band of 20–80 Hz was applied and the highest peak detected using the peak detection algorithm was determined as AVC.

Mitral valve opening: Finally, a search window with duration of 15% of the heart cycle and starting at the detected aortic valve closure was used to locate the last point of interest. A 3rd order Butterworth lowpass filter with cutoff at 15 Hz was applied and the first dip in the signal following the AVC detected using the peak/valley detection algorithm was determined as MVO.

An automatic error-checking algorithm was developed to remove beats where the detected time-points were regarded as incorrect based on statistical consistency: A moving average and standard deviation of time to R-peak for each of the four detected events was calculated using the previous five beats. If any of the four detected events in the current beat had a time to R-peak outside the range of the moving standard deviation, the detected event was considered a false positive and the beat discarded from further analysis. Finally, to check the accuracy of our algorithm, the time difference was calculated between the hemodynamically defined reference points and the valve events detected automatically by the algorithm.

#### Estimating the left ventricular pressure trace

Estimation of the LV pressure time trace was performed by adapting the method by Russell et al*.*^10^ to our measurements with the automatically detected valve events. The animation in online supplement #1 shows the procedure to create the normalized, average LV pressure trace as described here: The normalized, averaged LV pressure trace was calculated by pooling single cycle LV pressure traces from the canine experiments during baseline. These individual LV pressure traces were normalized using timing of valve events, found from the accelerometer signal processing algorithm above, in the following steps:

(i) Marking the time-points of opening and closing of the mitral and aortic valves in each of the individual LV pressure traces.

(ii) Stretching or compression of each of the raw pressure traces along the time-axis between valve events so that the opening and closing of valves aligned for all individual traces. The length of each heart cycle was arbitrarily set as 700 ms with MVC as the starting point (0 ms). The time-points of AVO, AVC, and MVO were fixed at 75 ms, 325 ms, and 400 ms, respectively.

(iii) Amplitude scaling of all the modified pressure traces to have the same peak value, arbitrarily chosen to 120 mmHg.

(iv) An averaged trace was then calculated using all these modified pressure traces (Fig. 5).

In order to estimate an individual LV pressure trace for a given heartbeat in any of the animals, the process was reversed. The animation in online supplement #2 shows the procedure as described here: The amplitude of the normalized, averaged pressure trace was first modified to match the measured peak LV pressure of the heartbeat. The isovolumic contraction, ejection, isovolumic relaxation, and filling phases of the trace were then stretched or compressed along the time-axis to match with the corresponding intervals found from the automatically detected valve events by the accelerometer for the given heartbeat. All the data from both the canine and porcine experiments were used to validate the estimated left ventricle pressure traces. The difference between the estimated LV pressure trace and the measured LV pressure trace was assessed for each individual heart cycle.

#### Pressure–displacement loop

Displacement was calculated by integrating the measured circumferential acceleration once to velocity and twice to displacement. The integrations were performed for one heart cycle at the time. Due to the cyclic motion, a point on the myocardial surface will start and end in approximately the same position in space during each heart cycle. This implies that the mean acceleration and mean velocity over each heart cycle is zero. Due to the addition of gravity in the sensed acceleration, mean acceleration will generally not be zero. The average acceleration and average velocity over one cycle were therefore subtracted before the respective integration to remove the static gravity component. For simplicity displacement was extracted only along the circumferential direction. Pressure–displacement loops were then generated using the estimated LV pressure trace and the actual measured pressure trace for comparison.

The areas of the pressure–displacement loops generated using the estimated LV pressure and measured LV pressure trace were calculated and compared. This was done for both the canine (n = 20), open chest porcine (n = 83) and closed chest porcine (n = 27) interventions.

### Statistical analysis

All statistical calculations were performed using R v3.4.1. For pressure–displacement loop area, variables were compared using least-squares linear regression and Pearson’s correlation coefficients. To account for multiple measurements from each animal when using the Bland–Altman plots, reference intervals were displayed based on agreement between methods of measurement with multiple observations per subject^24^. To test if the method had comparable performance in closed and open chest settings, a paired t-test was applied to investigate if there was a difference in the correlation coefficient of the estimated pressure or the error in the pressure–displacement loop area in animals where recordings were performed in both settings.

## Results

### Event detection

**Figure 4:**
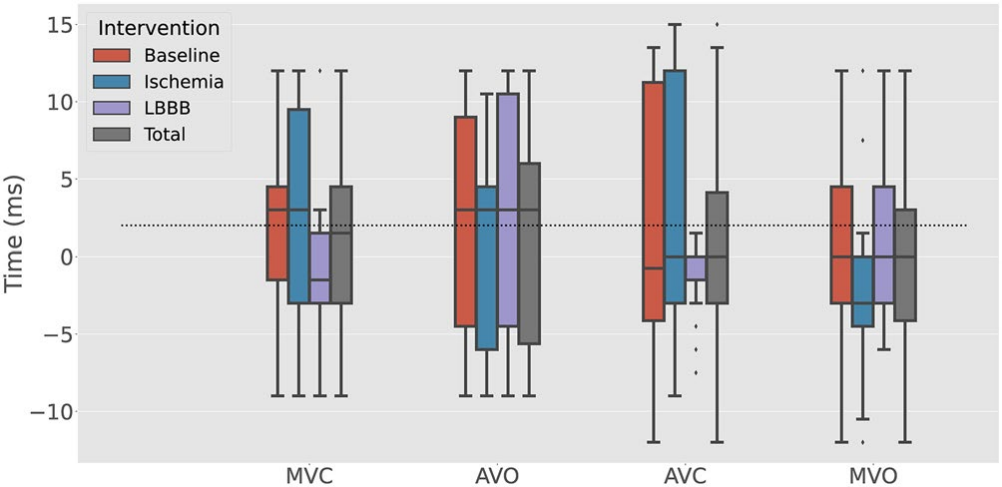
Box plot showing the difference between the accelerometer-detected and hemodynamically defined valve events for the three interventions in the canine study. Grey boxes show the total pooled results for all the three interventions and the dotted line represents the median of all the pooled data (2 ms). *LBBB* left bundle branch block, *MVC* mitral valve closure, *AVO* aortic valve opening, *AVC* aortic valve closure, *MVO* mitral valve opening.

The valve detection algorithm was run on a total of 20 canine recordings over a span of 20 heart beats each. The time difference between the automatically detected valve events and the hemodynamically defined events is shown in Fig. 4. The median difference and interquartile range for MVC, AVO, AVC and MVO were 2 ms [− 3, 4.5], 3 ms [− 6, 6], 1 ms [− 3, 4], and 0 ms [− 4.5, 3] respectively, for all 3 interventions pooled together. Overall, the algorithm detected all four valve events in 85% of the total 2400 analyzed heartbeats from both the canine and porcine experiments.

### Estimated left ventricular pressure

**Figure 5:**
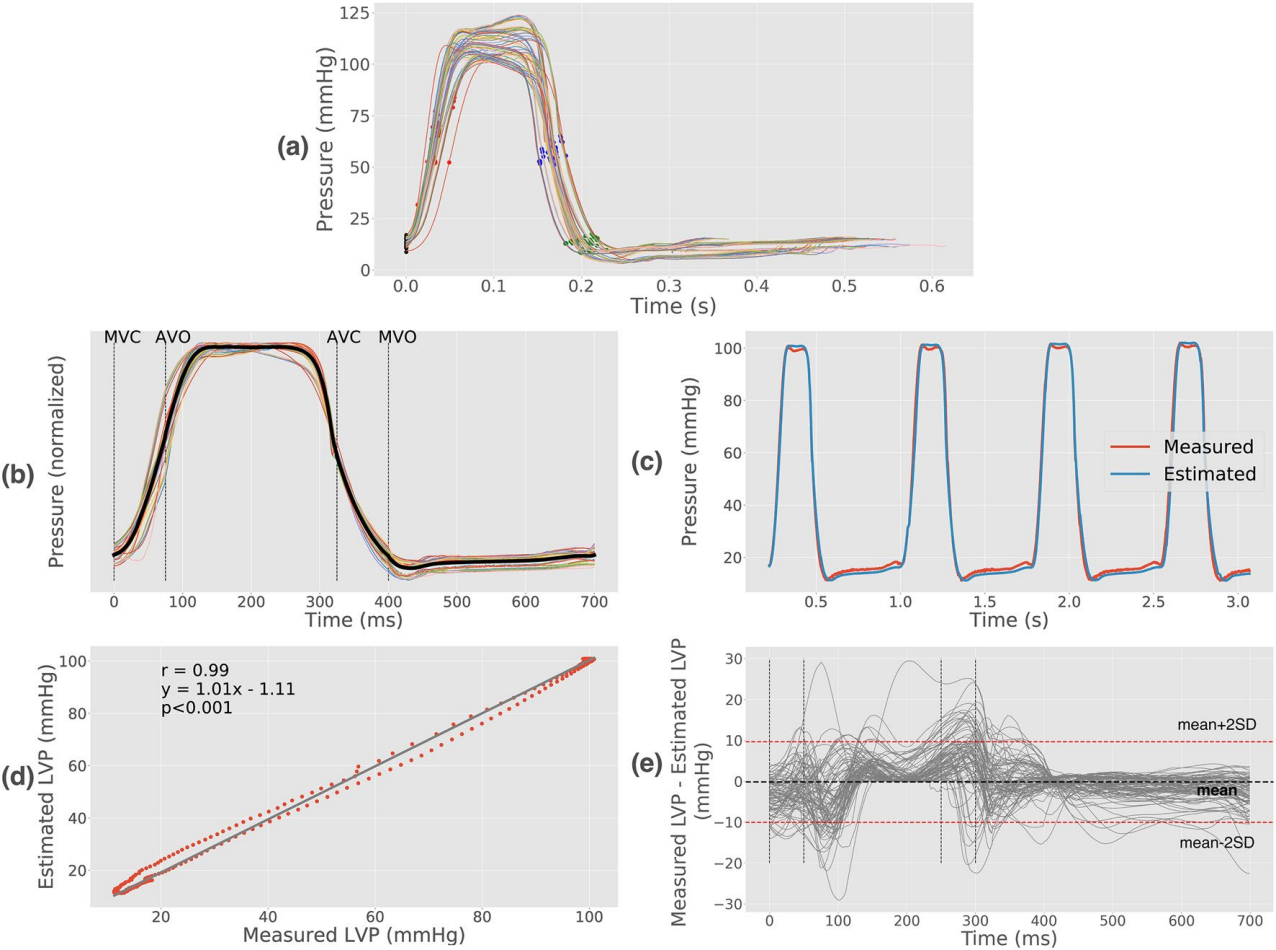
**(a)** Individual left ventricular (LV) pressure traces used to calculate the normalized, averaged pressure trace. Mitral and aortic valve events are indicated. **(b)** Traces from **(a)** adjusted along the time and pressure axes. The resulting normalized, averaged LV pressure trace shown with thick black line. **(c)** Comparison between measured and estimated LV pressure from a representative example. **(d)** Correlation between the estimated and measured trace shown in **(c)**. **(e)** Difference between measured and estimated LV pressure over time for all recordings. *LVP* left ventricular pressure, *MVC* mitral valve closure, *AVO* aortic valve opening, *AVC* aortic valve closure, *MVO* mitral valve opening.

The resulting normalized, averaged LV pressure trace is shown in Fig. 5b together with the individual pressure traces from the ten baseline canine recordings. There was a very good agreement between the estimated and measured LV pressure trace as seen in the representative case in Fig. 5c,d. For all 130 cases (20 from the canine studies, 83 from the open chest porcines studies and 27 from closed chest porcine studies), the average correlation coefficient was r = 0.98 and the limits of agreement − 10 to 10 mmHg (Fig. 5e). The highest difference between the estimated and measured LVP was seen around AVO. Peak LV pressure was 92 ± 12 mmHg in baseline, while ranging from minimum during infusion of beta blocker (75 ± 8 mmHg) to maximum during infusion of adrenaline (145 ± 15 mmHg).

### Pressure–displacement loops

**Figure 6:**
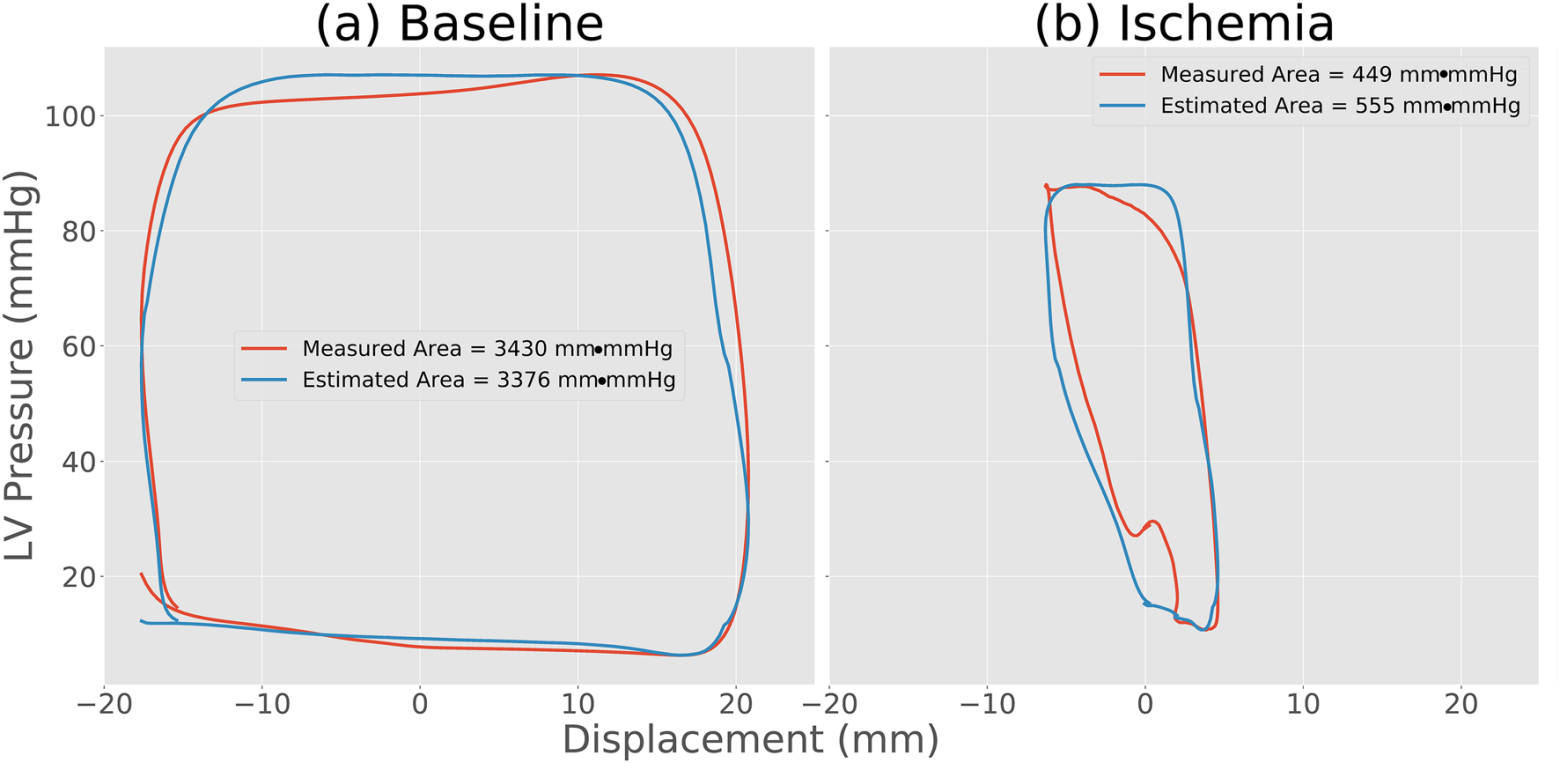
Comparison of pressure–displacement loops at baseline and during ischemia in a representative animal using estimated (blue) and measured pressure traces (red). The “bulge” in the lower left corner of the red loop during ischemia is presumably due to a measurement artifact caused by the pressure catheter coming in contact with the ventricular wall.

**Figure 7:**
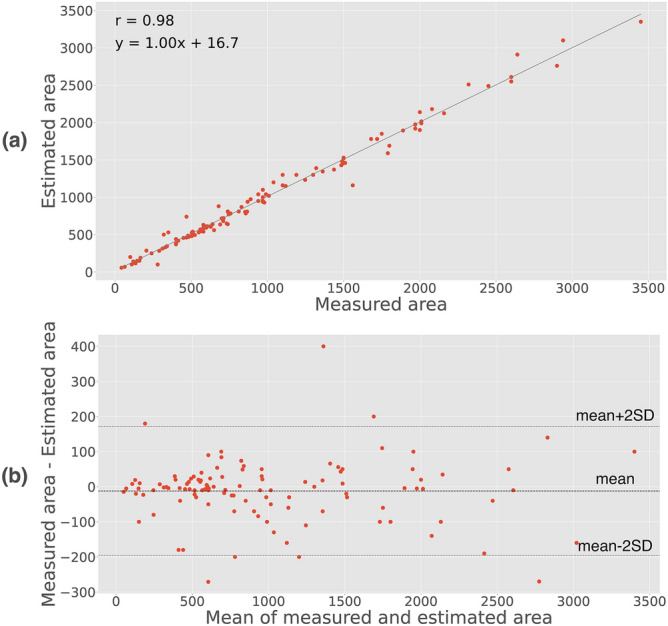
**(a)** Correlation and **(b)** Bland–Altman plot for pressure–displacement loop areas using measured vs. estimated pressure. Area is shown in mm·mmHg.

Figure 6 compares LV pressure–displacement loops using both estimated and measured pressure traces in a representative animal during baseline and ischemia. As can be seen in Fig. 6b, there was a “bulge” in the lower left corner of the loop generated using the measured LV pressure trace. This was presumably due to an artifact in the measured pressure trace which may occur in cases when the pressure catheter comes in contact with the wall during the heart cycle. Such artifacts were observed in 20 of the 130 recordings, which may have increased the difference between the estimated and measured data. Hence, the actual accuracy may be slightly better than the reported results. For all 130 cases, the correlation between pressure–displacement loop area using estimated vs. measured LV pressure traces was r = 0.98 (Fig. 7a). The mean difference was 10(± 186 2SD) mm·mmHg (Fig. 7b).

### Comparison of estimated pressure under open and closed chest conditions

**Figure 8:**
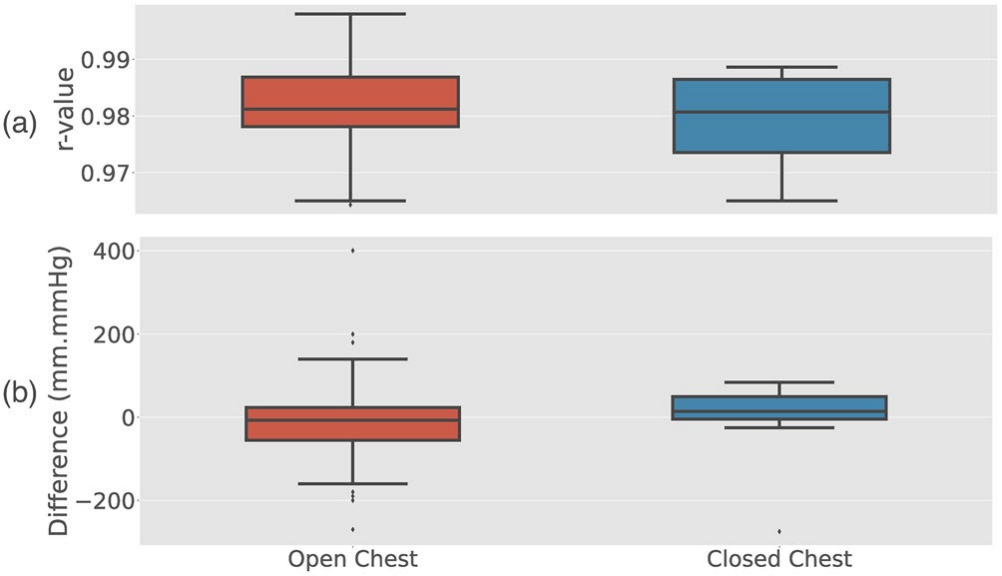
Comparison of the correlation coefficient (r-value) of the estimated pressure **(a)**, and the error in estimated pressure–displacement loop area **(b)**, between open (n = 103) and closed chest (n = 27) animal recordings.

The paired t-test between open and closed chest porcines during baseline (n = 8) did not show a significant difference neither for the accuracy of estimated pressure (p = 0.31 for the correlation coefficient) or for the error in estimated pressure–displacement loop area (p = 0.30). When pooling all open chest results (n = 103) and comparing it to the pooled closed chest results (n = 27), the overlapping ranges of the correlation coefficient between measured and estimated LV pressure and pressure–displacement loop area error suggest that the estimation method works in both the open and closed chest setting (Fig. 8).

## Discussion

In this paper we have proposed an algorithm for automatic detection of the opening and closing of the mitral and aortic valves by using an accelerometer attached to the heart. These detected valve events were used to estimate the LV pressure trace for each heartbeat. This was accomplished by adjusting a normalized, averaged LV pressure trace that had marked timings of the valve events. The isovolumic contraction, ejection, isovolumic relaxation, and filling phases of this trace were adjusted to the detected valve events for the individual heartbeat to generate the estimated LV pressure trace. Finally, using the displacement trace obtained from integrating the accelerometer signal twice, a pressure–displacement loop was obtained. The pressure–displacement loop area has been proposed as a parameter that reflects LV function well^9^. By our proposed method, it is now possible to obtain this pressure–displacement loop area solely from accelerometer recordings, without measuring LV pressure. This was supported by the good accuracy of detecting the valve events and estimating the LV pressure and loop area.

While the purpose of detecting valve events in this study was to estimate the LV pressure trace and pressure–displacement loop area, assessment of these events per se can be useful to derive other functional indices. For example, the myocardial performance index, also known as the Tei Index, is a pure time index that can be directly calculated from these detected events as the sum of the duration of the isovolumic periods divided by ejection time^25^. Furthermore, post-systolic shortening is a characteristic sign of myocardial dysfunction during ischemia^26^. This abnormal post systolic motion is evident also in velocity measurements^27^ and in our experience the abnormal post-systolic velocity wave is present in the velocity trace from the integrated accelerometer signal as well. Hence, automatic detection of aortic valve closure will now facilitate automatic extraction of such post-systolic functional indices, potentially improving automatic monitoring of the cardiac function by accelerometers. The ability to reflect cardiac function of other such indices in addition to the proposed pressure–displacement loop area method, should be investigated in future studies.

The detection algorithm was based on first detecting the ECG R-peaks to segment the acceleration trace into individual heart cycles. ECG is in most cases recorded and available for such R-peak detection. However, the acceleration signal itself can be used to detect end diastole and segment the individual heart cycles^28^, which can be used in combination with ECG to make the detection more robust or as a standalone method to simplify the monitoring system and reduce the need for additional ECG leads and hardware. All digital filters used in the detection algorithm were applied as two-way zero-phase filters, applied both in the forward and reverse directions. This avoids phase shifts and time-delays, ensuring optimal interpretation of the data in a research setting. However, this is only possible when post-processing recorded data. Real-time operation will require an alternative implementation of these filters, finding a balanced optimum between response time and signal quality.

The normalized, averaged LV pressure trace in this study was generated using the data from baseline canine recordings only. This trace was then used to estimate the LV pressure trace for all interventions in both the canine and porcine data. Judging from the correlation and Bland–Altman plot in Fig. 7 this pressure trace worked generally well, suggesting that the exact shape of the pressure trace is less critical. This is consistent with the error analysis in the study by Russell et al*.* which showed relatively small changes in loop area with respect to the shape of the pressure trace during ejection and ± 30 ms variations in timing of AVC and AVO^10^.

In order to estimate the individual pressure trace, the normalized, averaged trace must be scaled both along the time-axis and pressure-axis. The automatic detection of valve events allows automatic scaling along the time-axis. Scaling of peak pressure may be done manually as it can be approximated as the brachial cuff pressure in the absence of large pressure gradients across the aortic valve or pressure differences to this more distal artery. In cases with continuous recordings of arterial pressure, one could envision that the estimated LV pressure can be automatically scaled based on input from such measurements. While maximum pressure during ejection may be relatively easy to scale to the individual, the pressure during diastolic filling can currently not be scaled in a similar manner due to lack of measurements of this diastolic LV pressure. Thus, the pressure–displacement loop area will be overestimated in cases where LV filling pressure is abnormally high. To indicate the sensitivity of the estimated loop area to errors in either estimated peak systolic or ventricular filling pressure, we may approximate the loop as a rectangle. In such rectangular case where the ventricular pulse pressure is 100 mmHg, a 1 mmHg error in maximum or minimum pressure, causes a 1% error in loop area.

We did not have reference measurements for timing of valve events under closed chest conditions, so we were unable to evaluate if the accuracy of the detection of these events is similar under closed- vs. open chest conditions. However, we were able to assess the accuracies of both the estimated pressure time-trace and pressure–displacement loop area in animals with closed chest, which were comparable to the accuracies in open chest and therefore indicate that the presented method also works in closed chest settings.

The number of animals in each intervention was relatively low, and the study was not designed to investigate if there were significant differences in the accuracy of the method between these interventions. Rather it was a proof of concept study where we applied the method on available retrospective data. We did not observe large outliers from any of the interventions, and based on the observed error ranges, potential difference in errors between interventions seemed negligible for practical purposes. However, the results are only from a few interventions in a limited number of animals, and do not guarantee that the method will work in all situations.

## Conclusion

The signal from an epicardially attached accelerometer could be used for automatic detection of the time-points of mitral and aortic valve openings and closings with very good accuracy. These detected time-events could subsequently be used to estimate the LV pressure trace by scaling a normalized, averaged pressure trace with these marked events along its time-axis. Hence, the pressure–displacement loop area could be derived solely from accelerometer recordings with very good accuracy. The results encourage further investigations on how the developed methods can be used to improve monitoring of cardiac function by accelerometers.

## Supplementary information

Supplementary Video 1.

Supplementary Video 2.
